# Comprehensive Analysis and Functional Verification of the *Pinus massoniana* NBS-LRR Gene Family Involved in the Resistance to *Bursaphelenchus xylophilus*

**DOI:** 10.3390/ijms24031812

**Published:** 2023-01-17

**Authors:** Yini Xie, Bin Liu, Kai Gao, Yunxiao Zhao, Wenhua Li, Lili Deng, Zhichun Zhou, Qinghua Liu

**Affiliations:** 1 Research Institute of Subtropical Forestry, Chinese Academy of Forestry, Hangzhou 311400, China; 2 Faculty of Forestry, Nanjing Forestry University, Nanjing 210037, China; 3 Zhejiang Provincial Key Laboratory of Tree Breeding, Hangzhou 311400, China

**Keywords:** NBS-LRR gene family, disease resistance genes, *Pinus massoniana*, pine wood nematode, *PmNBS-LRR97*, ROS

## Abstract

*Pinus massoniana* Lamb. is a crucial timber and resin conifer in China, but its plantation industry is threatened by outbreaks of pine wilt disease (PWD) caused by *Bursaphelenchus xylophilus* (pinewood nematode; PWN). However, as of yet, there is no comprehensive analysis of NBS-LRR genes in *P. massoniana* involved in its defense against PWN. In this study, 507 NBS genes were identified in the transcriptome of resistant and susceptible *P. masoniana* inoculated with the PWN. The phylogenetic analysis and expression profiles of resistant and susceptible *P. massoniana* revealed that the up-regulated *PmNBS-LRR97* gene was involved in conferring resistance to PWN. The results of real-time quantitative PCR (qRT-PCR) showed that *PmNBS-LRR97* was significantly up-regulated after PWN infection, especially in the stems. Subcellular localization indicated that PmNBS-LRR97 located to the cell membrane. *PmNBS-LRR97* significantly activated the expression of reactive oxygen species (ROS)-related genes in *P. massoniana*. In addition, the overexpression of *PmNBS-LRR97* was capable of promoting the production of ROS, aiding in plant growth and development. In summary, *PmNBS-LRR97* participates in the defense response to PWN and plays an active role in conferring resistance in *P. massoniana*. This finding provides new insight into the regulatory mechanism of the R gene in *P. massoniana*.

## 1. Introduction

Plants are subjected to external biotic and abiotic stresses throughout their life cycle. Over the course of long-term evolution, a complete immune system has evolved to resist such external threats to plant fitness. Two defense mechanisms, pathogen-associated molecular pattern (PAMP)-triggered Immunity (PTI) and effector-triggered immunity (ETI), constitute the immune system of plants. When plants are attacked by pathogens, pattern recognition receptors (PRRs) on the cell surface identify PAMPs from attacking species and trigger successive defense responses. However, in the process of host–pathogen interaction, the effector proteins secreted by the invading pathogen can inhibit PTI and destroy the first line of defense against entry into plant cells. At this time, the intracellular immune receptor recognizes the effector proteins that enter the cells and triggers a defense response [1,2,3,4]. Pattern recognition receptors in PTI and intracellular immune receptors in ETI are collectively referred to as R genes, which can recognize pathogens, carry out signal transduction, and cause downstream defense responses, collectively rendering plants resistant to their enemies [5]. Up to now, more than 400 disease R genes have been cloned from different plants, and nine molecular mechanisms of R gene-mediated resistance have been identified [6,7].

The NBS-LRR gene family is the most numerous and widely distributed category of R genes, encoded proteins that are mainly composed of a nucleotide binding site (NBS) and a leucine-rich repeat (LRR) region [8]. The NBS is a principal domain coded by the NBS-LRR gene and includes eight conserved motifs: P-loop, RNBS-A, Kinase2, RNBS-B, RNBS-C, GLPL, RNBS-D, and MHDV. The NBS is primarily responsible for the binding and hydrolysis of ATP and GTP. The LRR domain can specifically recognize pathogens and interact with effector proteins. The N-terminal domain is involved in pathogen recognition and the initial transduction of disease resistance signals [9,10]. The NBS-LRR gene family has been analyzed in *Arabidopsis thaliana* (L.) Heynh. [11], *Populus trichocarpa* Torr. & Gray [12], *Eucalyptus grandis* Hill ex Maiden [13], *Oryza sativa* L. [14], *Glyine max* (Linn.) Merr. [15], *Solanum tuberosum* L. [16], and some other plant species. The Hs1pro-1 gene was the first R gene cloned from sugar beet (*Beta vulgaris* L.), which was resistant to the beet cyst nematode (*Heteroderaschachtii*) [17]. The R genes (*Xa21* and *Xa26*) in O. sativa play a vital role in its resistance to *Xanthomonas oryzae* pv. *oryzae*, and the overexpression of *Xa21* and *Xa26* conferred stronger resistance [18,19]. A TIR-NBS-LRR gene, *GmKR3*, was identified in *G. max*, and its overexpression can augment the resistance to soybean mosaic virus (SMV), bean common mosaic virus (BCMV), watermelon mosaic virus (WMV), the secovirus bean pod mottle virus (BPMV), and other virus strains, without affecting plant growth and development [20].

*Pinus massoniana* Lamb. is an indispensable industrial timber and resin tree species native to China, where it is a pivotal forest resource [21]. Due to its high output of turpentine, it is widely used in the rubber industry, paper industry, soap industry, perfume industry, and adhesive industry, among others. Pine wilt disease (PWD) is a globally devastating disease caused by *Bursaphelenchus xylophilus* (pinewood nematode; PWN), and it is currently also the most severe forest disease facing pine forests in general [22], which seriously threatens the ecological and economic security of not only Asia [23,24,25] but also Europe [26,27] and North America [28] and perhaps even the world entirely [29,30]. Although the functions of some resistance-related genes are known, such as those of *PmGPPS1* [31], *PmTPS4*, *PmTPS21* [32], and *PmCYP720B11v2* [33], the related functions of R genes are rarely reported.

The lack of an in-depth analysis of resistant mechanisms limits our knowledge of defense towards PWN. Hence, the verification of R gene functions could provide a new perspective for analyzing the mechanism underpinning *P. massoniana* resistance to PWN. Accordingly, in this study, the members of the NBS-LRR gene family in the transcriptome of *P. massoniana* were identified, grouped, and structurally analyzed using bioinformatic techniques, from which the differentially expressed gene *PmNBS-LRR97* was cloned. Its sequence, expression pattern, and functions were analyzed, thus laying a theoretical foundation for analyzing the molecular mechanism by which *P. massoniana* resists PWN.

## 2. Results

### 2.1. Identification of NBS Genes in P. massoniana

A total of 507 NBS proteins were obtained from the *P. massoniana* transcriptome (Table 1 and Table S1). Based on the domain predictions, the sequences were divided into eight subclasses: N (NBS), NL (NBS-LRR), TNL (TIR-NBS-LRR), TN (TIR-NBS), CNL (CC-NBS-LRR), CN (CC-NBS), RNL (RPW8-NBS-LRR), and RN (RPW8-NBS). The TNL subclass had the largest number of genes, 120, accounting for 23.67% of the total, whereas the CN subclass had the fewest, only 6, or 1.18% of the total. Four types of genes contained an LRR domain, totaling 292 genes (57.59% of the total), while four types of genes lacked an LRR domain, for a total of 215. The total number and subclass number of NBS-LRR genes varied greatly among different plants. There were 402 NBS genes in *P. trichocarpa*, 105 fewer than those in *P. massoniana*, and 209 in *A. thaliana*, the latter accounting for just 41.22% of the total number present in *P. massoniana*. Like *P. massoniana*, TNL also had the most genes in *A. thaliana*, while in *P. trichocarpa*, the most abundant subclasses were NL and CNL; likewise, the least abundant subclasses among the three species also differed. They were CN, N, and TN. In addition, there were 31 other NBS genes in A. thaliana, and this subclass contained two types, TX (TIR-X) and C (CC (related to CNL)). Of course, the three species had similarities as well; for example, compared with the other subclasses, the CN subclass was relatively less represented in all three species.

### 2.2. Sequence, Phylogenetic, and Conserved Motif Analyses of NBS-LRR Resistance Genes in P. massoniana

The basic physicochemical properties, subcellular localization, presence or absence of signal peptides, and transmembrane domains of the protein sequences encoded by 292 NBS-LRR genes of NL, TNL, CNL, and RNL were analyzed (Table S2).

**Figure 1:**
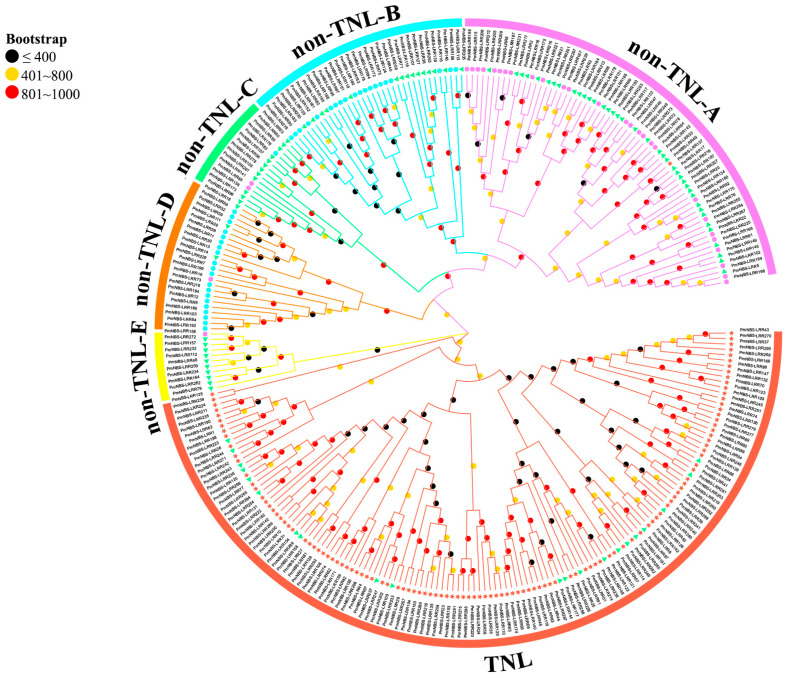
Phylogenetic tree of *P. massoniana* NBS-LRR disease resistance genes based on NBS domain regions. A maximum likelihood (ML) phylogenetic tree with 1000 bootstrap repeats was constructed with MEGA7.0. Stars, triangles, circles, and squares represent TNL, NL, RNL, and CNL predicted proteins, respectively. The bootstrap values are represented by different colored circles on the branches.

The NBS domain region sequences were extracted from 292 NBS-LRR genes, and MEGA7.0 was used for their phylogenetic analysis. The resulting phylogenetic tree shows that the 292 NBS-LRR disease resistance proteins could be clearly divided into two large branches (Figure 1): TNL and non-TNL, with the non-TNL branch further divided into five small branches (non-TNL-A, non-TNL-B, non-TNL-C, non-TNL-D, and non-TNL-E). TNL subclass genes are basically homogenous in the large TNL branch. The NL subtype genes were distributed in the two large branches, with more genes in the non-TNL large branch than in the TNL large branch, indicating that the origin of these subtypes’ genes was more diverse. The genes of each subclass in the non-TNL large branch were not clustered on one branch as clearly as the TNL subclass genes were. The NL subclass genes were mainly clustered in the non-TNL-C and non-TNL-E small branches. The genes of RNL subtypes were mainly clustered in the non-TNL-B and non-TNL-D branches, and the CNL subtype genes were mainly clustered in the non-TNL-A branch. Apart from most genes of each subtype being clustered in one branch, there were a few genes scattered in other small branches.

The conserved motifs of 292 NBS-LRR genes were analyzed using the MEME online software. This revealed that the composition and arrangement of the conserved domains and motifs of genes in the same clade were basically the same, whereas the differences between different clades were pronounced (Table S3 and Figure S1). There was little difference between TNL subclass genes, with 4, 10, and 7 motifs found in the TIR, NBS, and LRR domains, respectively. The seven motifs of the LRR domain were named L1-7, respectively. Compared with the TIR and NBS domains, the structure of the LRR domain was relatively less conserved. Due to the large sequence differences among NL subtype genes, they had multiple origins; hence, the composition and arrangement of motifs differed markedly between different small branches. A special motif (motif 24), named RPW8-1, was found in the RPW8 domain of RNL subclass genes. No motifs were found in the CC domain of CNL subclass genes, probably because the N-terminal sequence was not highly conserved. There was a motif (motif 19), named NBS-4, in the NBS domain of CNL subclass genes, which was not present in TNL subclass genes. Compared with the CC domain, the NBS domain was evidently highly conserved.

### 2.3. Differential Gene Expression Pattern of NBS-LRR and Cloning of PmNBS-LRR97 in P. massoniana

**Figure 2:**
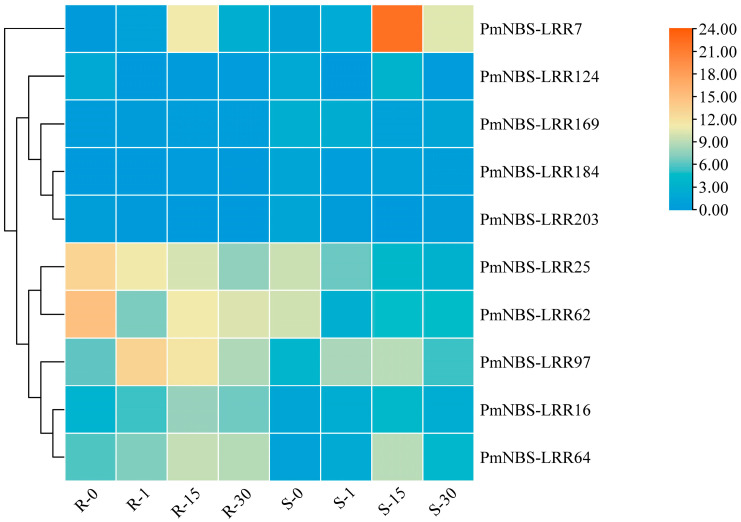
Expression patterns of differential NBS-LRR genes in different periods of resistant and susceptible *P. massoniana* inoculated with the PWN. High expression levels and low expression levels were represented by orange and blue, respectively. Differential genes were hierarchically clustered according to their expression and divided into different gene clusters in the graph.

A heat map was drawn using the expression data of differentially expressed NBS-LRR genes in the transcriptome of resistant versus susceptible *P. massoniana* at different days since the inoculation with the PWN (Figure 2). These results showed that the expression levels of *PmNBS-LRR25*, *PmNBS-LRR62*, *PmNBS-LRR97*, *PmNBS-LRR16*, and *PmNBS-LRR64* were greater in resistant than susceptible *P. massoniana*, all of which were up-regulated genes. Conversely, *PmNBS-LRR7*, *PmNBS-LRR124*, *PmNBS-LRR169*, *PmNBS-LRR184*, and *PmNBS-LRR203* were down-regulated genes whose expression levels in susceptible *P. massoniana* surpassed those in resistant *P. massoniana*. Further, the expression levels of *PmNBS-LRR25* and *PmNBS-LRR62* were decreased in both resistant and susceptible *P. massoniana* after the inoculation with the PWN, while those of *PmNBS-LRR97*, *PmNBS-LRR16*, and *PmNBS-LRR64* increased by 2.17-fold, 1.66-fold, and 1.21-fold in resistant *P. massoniana*. Accordingly, *PmNBS-LRR97* was regarded as the dominant gene involved in PWN infection.

**Figure 3:**
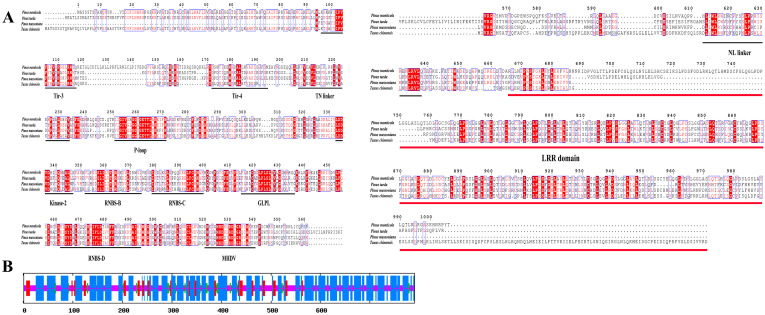
Sequence and protein structure analysis of *PmNBS-LRR97* of *P. massoniana*. (**A**) Analysis of the conserved domains and conserved motifs of TNL homologous genes in *P. massoniana*, *P. monticola*, *P. taeda*, and *Taxus chinensis*. (**B**) Prediction of the secondary structure of the *PmNBS-LRR97*. Blue, red, green, and orange represent alpha helix, extended strand, beta turn, and random coil, respectively.

Sequence analysis showed that the *PmNBS-LRR97* gene sequences in resistant and susceptible *P. massoniana* were entirely identical. The full-length cDNA of the sequence was 2373 bp, encoding a total of 790 aa (Figure 3A), with a molecular weight of 90.213 kDa and an isoelectric point of 6.18. It is an unstable protein given its instability index of 98.04; it is a thermophilic protein whose grand average for hydrophilicity is less than 0, meaning that it is also a hydrophilic protein; it lacks both a signal peptide and a transmembrane domain; and it contains 61 phosphorylation sites.

The *PmNBS-LRR97* sequence contained TIR, NBS, and LRR domains and was evidently a TNL subclass gene. An analysis of its secondary structure showed that the PmNBS-LRR97-encoded protein had four structural elements: an alpha helix, an extended strand, a beta turn, and a random coil (Figure 3B). Among them, the alpha helix is composed of 448 aa, accounting for the most amino acids (56.71%), while the beta turn consisted of 28 aa, the fewest (3.54%).

### 2.4. Expression Pattern of PmNBS-LRR97 in Resistant and Susceptible P. massoniana

**Figure 4:**
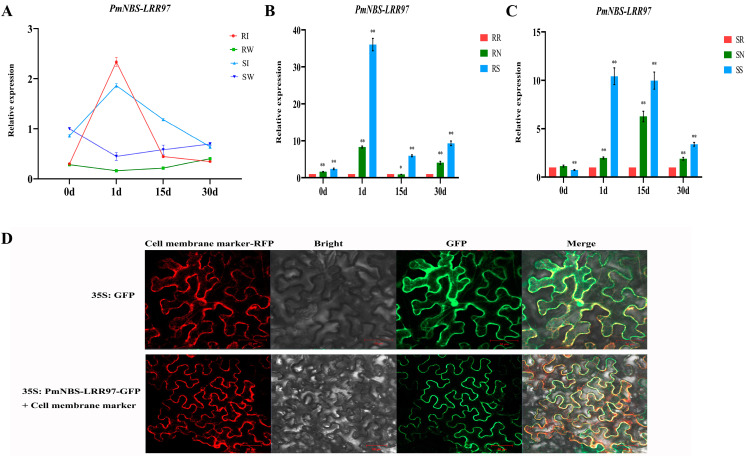
Expression pattern and subcellular localization of *PmNBS-LRR97* in resistant and susceptible *P. massoniana*. (**A**) Expression patterns of *PmNBS-LRR97* in resistant and susceptible *P. massoniana* inoculated with the PWN at 0, 1, 15, and 30 dpi. Error bars represent three biological replicates ± SD. RI and RW represent resistant *P. massoniana* inoculated with the PWN and water, respectively; SI and SW represent susceptible *P. massoniana* inoculated with the PWN and water, respectively. Susceptible *P. massoniana* at 0 days of water inoculation served as a control. (**B**) The expression patterns of *PmNBS-LRR97* in different tissues at different stages of the resistant *P. massoniana* inoculated with the PWN. Error bars represent three biological replicates ± SD. RR, RN, and RS represent the root, stem, and needle of resistant *P. massoniana*, respectively. The root of resistant *P. massoniana* was used as the control in each period. (**C**) The expression patterns of *PmNBS-LRR97* in different tissues at different stages of susceptible *P. massoniana* inoculated with the PWN. Error bars represent three biological replicates ± SD. SR, SN, and SS represent the root, stem, and needle of susceptible *P. massoniana*, respectively. The root of susceptible *P. massoniana* was used as the control in each period. Student’s *t*-test was used to test the significance (*, *p* < 0.05; **, *p* < 0.01). (**D**) Subcellular localization of the PmNBS-LRR97. Empty vector containing the 35S promoter and PmNBS-LRR97 co-localized with cell membrane markers, respectively. Scale bar = 50 μm.

Taking the susceptible *P. massoniana* inoculated with water at 0 days post-inoculation (dpi) as the control, the expression pattern of *PmNBS-LRR97* in the stems of resistant and susceptible *P. massoniana* inoculated with the PWN was analyzed by qRT-PCR at 0, 1, 15, and 30 dpi (Figure 4A). At each time point, after inoculating the resistant and susceptible *P. massoniana* with water, the gene’s expression level basically did not exceed that at 0 dpi; in stark contrast, after resistant and susceptible *P. massoniana* were inoculated with PWN, the gene featured an ‘up-down-down’ expression trend. Compared with the respective expression levels at 0 dpi, the expression level of resistant *P. massoniana* after inoculation was six times higher than that of susceptible *P. massoniana*. Next, the expression pattern of *PmNBS-LRR97* in different tissues was explored by comparing the roots of resistant and susceptible *P. massoniana* exposed to PWN at different times post-inoculation (Figure 4B,C). The gene’s expression level in stems was higher than that in roots or needles across all time points, regardless of whether *P. massoniana* was resistant or susceptible.

The subcellular localization results showed that PmNBS-LRR97 located to the cell membrane (Figure 4D), which is consistent with the function of the R gene of recognizing effectors.

### 2.5. Overexpression of PmNBS-LRR97 Enhances Plant Growth and Increases the Content of Defense Signaling Molecules

**Figure 5:**
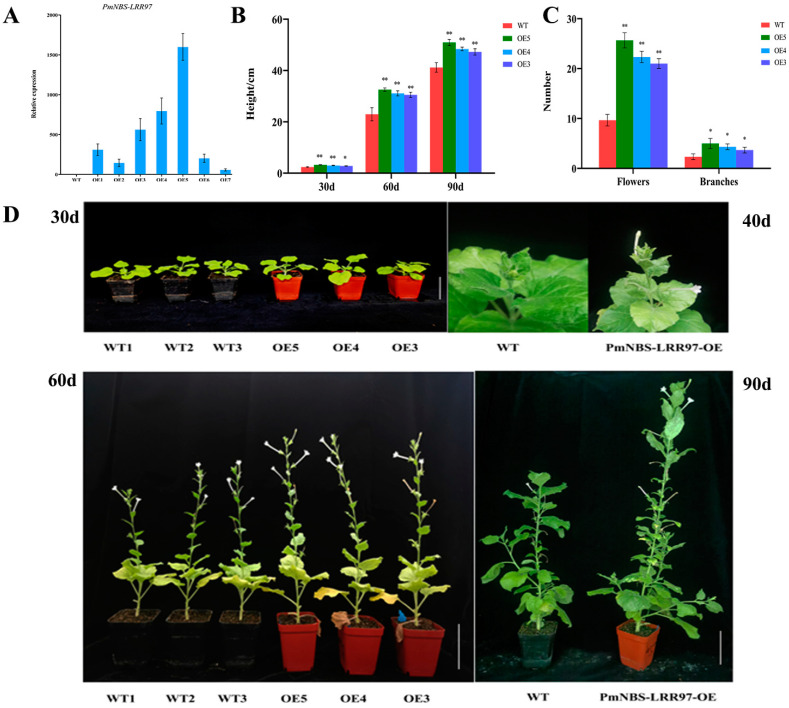
Quantitative verification and phenotypic difference analysis of *PmNBS-LRR97*-overexpressing tobacco. (**A**) Relative expression of *PmNBS-LRR97* in WT and transgenic tobacco; error bars represent three biological replicates ± SD. (**B**) Plant height analysis of WT and *PmNBS-LRR97*-overexpressing tobacco at 30, 60, and 90 d; error bars represent three biological replicates ± SD. Student’s *t*-test was used to test the significance (*, *p* < 0.05; **, *p* < 0.01). (**C**) Differences in the flowering number and branch number between WT and *PmNBS-LRR97*-overexpressing tobacco at 90 d; error bars represent three biological replicates ± SD. (**D**) Different phenotypes of WT and *PmNBS-LRR97*-overexpressing tobacco. Scale bar = 7 cm.

*PmNBS-LRR97* was transformed in *N. benthamiana* to verify its function, because the genetic transformation system of *P. massoniana* has not been successfully established. The qRT-PCR results demonstrated that, compared with WT, the *PmNBS-LRR97* expression levels of all transgenic tobacco lines were higher, with those of *PmNBS-LRR97*-OE5, *PmNBS-LRR97*-OE4, and *PmNBS-LRR97*-OE3 being the greatest; hence, these three lines were used for further verification (Figure 5A).

To explore whether *PmNBS-LRR97* can affect plant growth, the phenotypes of WT and transgenic tobacco were observed (Figure 5B–D). The transgenic tobacco plants were taller than WT counterparts, irrespective of their age (i.e., 30, 60, and 90 d) (*p* < 0.01). It was also found that the transgenic tobacco bloomed earlier than WT and had more flowers and greater branching at 90 days, indicating that the overexpression of *PmNBS-LRR97* can effectively promote the growth and development of plants.

**Figure 6:**
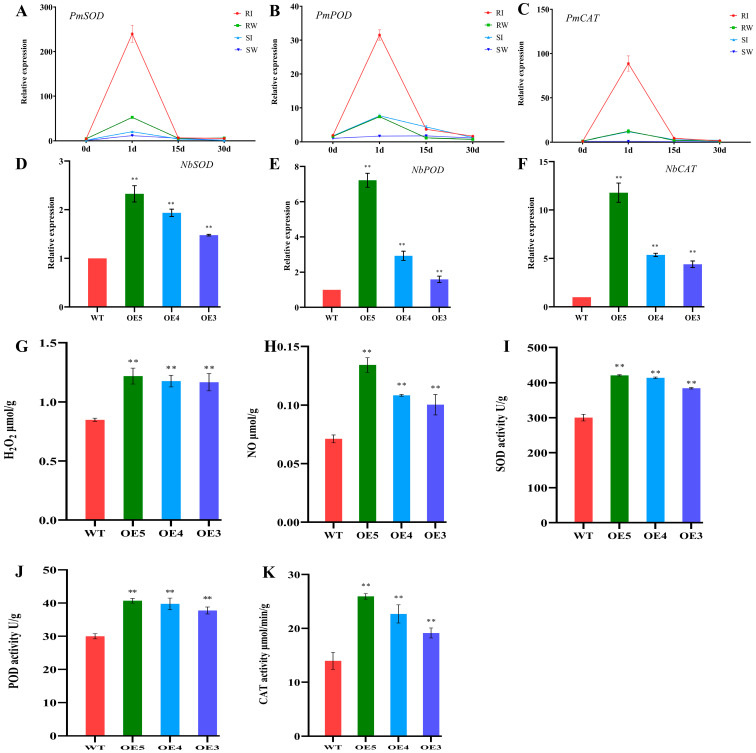
Physiological differences between *P. massoniana* and tobacco. (**A**–**C**) Expression patterns of *PmSOD*, *PmPOD*, and *PmCAT* in resistant and susceptible *P. massoniana* inoculated with the PWN at 0, 1, 15, and 30 dpi. Error bars represent three biological replicates ± SD. RI and RW represent resistant *P. massoniana* inoculated with the PWN and water, respectively; SI and SW represent susceptible *P. massoniana* inoculated with the PWN and water, respectively. Susceptible *P. massoniana* at 0 days of water inoculation served as a control. (**D**–**F**) Differences in the relative expression levels of *NbSOD*, *NbPOD*, and *NbCAT* in WT and transgenic tobacco. (**G**–**K**) The contents of H_2_O_2_, NO, SOD, POD, and CAT were different between WT and *PmNBS-LRR97*-overexpressing tobacco. Error bars represent three biological replicates ± SD. The difference was statistically significant by Student’s *t*-test analysis (**, *p* < 0.01).

Plant disease resistance genes can recognize effectors secreted by nematodes, activate signaling pathways, and cause a series of defense responses. The burst of reactive oxygen species (ROS) is the initial defense response. The production and scavenging of ROS in plants are normally maintained in a dynamic equilibrium [34], but once stimulated by foreign organisms, that dynamic balance will falter and cause damage to plants. The expression patterns of *PmSOD*, *PmPOD*, and *PmCAT* were analyzed in the stems of resistant and susceptible *P. massoniana* (Figure 6A–C) to explore the relationship between R genes and ROS-related genes. These results uncovered an ‘up-down-down’ expression trend for *PmSOD*, *PmPOD*, and *PmCAT* after inoculating the resistant and susceptible *P. massoniana* with the PWN. Moreover, the up-regulated fold-changes of *PmSOD*, *PmPOD*, and *PmCAT* were far greater in resistant than susceptible *P. massoniana*. This result was similar to the expression pattern of *PmNBS-LRR97*. Next, the expression of *NbSOD*, *NbPOD*, and *NbCAT* was analyzed in WT and transgenic tobacco (Figure 6D–F), and the contents of known plant defense signaling molecules (H_2_O_2_, NO) and enzymes related to the ROS scavenging system (SOD, POD, CAT) were detected in tobacco (Figure 6G–K). The expression levels of *NbSOD*, *NbPOD*, and *NbCAT* were all markedly higher in transgenic tobacco than WT, and the contents of H_2_O_2_, NO, SOD, POD, and CAT were also higher. This indicated that the overexpression of *PmNBS-LRR97* can increase the content of defense signal molecules and related enzymes, which is vital for the resistance of *P. massoniana* to the PWN.

## 3. Discussion

Plant R genes can specifically recognize effector proteins secreted by pathogens and initiate a cascade of downstream defense mechanisms, which is indispensable for plant resistance to pathogens [35,36,37]. The NBS-LRR gene family, the largest category of R genes, has been identified and systematically analyzed for its involvement in the defense processes of many plants. *P. massoniana*, as a pioneer conifer species, is vulnerable to the PWN. However, the defense strategy of R genes in *P. massoniana* facing an attack from the PWN remains unknown. Therefore, the identification, characterization, and functional analysis of R genes are crucial steps for understanding how *P. massoniana* is able to achieve resistance against PWN.

The members of the NBS-LRR gene family were identified in *P. massoniana* to gain insight into how they conferred resistance to the PWN. Given the differing genome sizes of plant species, their numbers of NBS-LRR gene families and subfamilies are also different. The results showed that 507 NBS genes were identified in the *P. massoniana* transcriptome by bioinformatic methods, more than those in *A. thaliana* [11] and *P. trichocarpa* [12] but fewer than those in *E. grandis* [13] or *O. sativa* [14]. The NBS-LRR gene family of *P. massoniana* can be separated into eight subclasses, of which the number of TNL subclass genes was 3.87 times that of CNL subclass genes; the number of TNL subclass genes in *A. thaliana* was 1.8 times that of CNL; and the number of CNL subclass genes in *P. trichocarpa* was 1.5 times that of TNL (Table 1). Different subtype genes occupy the main position in different plants, which may be caused by the long-term immune evolution of plants against pathogens. In short, different plants face different pathogens and thus likely rely on different resistance genes.

One phylogenetic tree of 292 NBS-LRR genes was built in *P. massoniana* (Figure 1). These 292 NBS-LRR genes divided neatly into two prominent branches (TNL and non-TNL), not unlike the classification reported for other plants [11,12,38,39,40,41]. It can be deduced from the phylogenetic tree results that the TNL-type genes evolved earlier than the non-TNL-type genes, which further supports the inference that TIR-type genes originated earlier than non-TIR-type genes [38,42]. Furthermore, the types of non-TNL genes are more diverse: CNL and RNL subclass genes clustered on the non-TNL branch, and NL subclass genes are distributed on each branch, indicating that NL-type genes have more diversified origins. The results of the gene conserved domain and conserved motif analyses (Table S3 and Figure S1) also demonstrated that genes within the same branch had similar conserved domains and conserved motifs, which strongly suggests that those genes have similar structures and functions. The NBS domain is the most conserved, while the N-terminal TIR/CC/RPW8 and C-terminal LRR domains are less conserved. Besides the conserved motifs of TIR-1-4, P-loop, Kinase2, RNBS-B, RNBS-C, GLPL, RNBS-D, and MHDV in the TNL subclass, the NBS domain lacks the RNBS-A motif identified in previous studies [43] but harbors three new conserved motifs, which may have been lost during the evolution of *P. massoniana* [44]. Compared with TNL subclass genes, the NL, RNL, and CNL subclass genes have a higher diversity in the arrangement and composition of domains and conserved motifs. Motif 19 and motif 24 are unique motifs in CNL and RNL subclass genes, respectively. This finding could be explained by the differential resistance genetic variation in *P. massoniana* in the face of different biotic or abiotic stresses. In summary, phylogenetic tree and conserved motif analyses provide compelling evidence for the complex evolutionary relationships of NBS-LRR gene family members and further explain the diversity of resistance in *P. massoniana*.

The expression profile of *PmNBS-LRR97* in the stem was analyzed to further clarify this gene’s involvement in conferring PWN resistance (Figure 4A). When a plant is not infected by one or more pathogens, its expression of the R gene is at a low level. Once infected by pathogens, the R gene will respond quickly, and its level of expression will increase rapidly [45,46,47]. Compared with the control group (inoculated with water), the expression of *PmNBS-LRR97* in resistant and susceptible *P. massoniana* was always greater at different times after the inoculation with the PWN, indicating that the inoculated wounds did not affect the expression of the *PmNBS-LRR97*. The expression of *PmNBS-LRR97* in resistant and susceptible *P. massoniana* increased after their inoculation with the PWN, but the fold increase was significantly higher in the former than in the latter, indicating that *PmNBS-LRR97* was linked to the resistance in *P. massoniana* to the PWN. At 15 dpi, this gene’s expression level in resistant *P. massoniana* basically reverted to its level before the inoculation, whereas in susceptible *P. massoniana*, it remained significantly higher. At 30 dpi, the expression of *PmNBS-LRR97* in resistant and susceptible *P. massoniana* was on par with that before their inoculation with the PWN.

We speculate that the *PmNBS-LRR97* of resistant *P. massoniana* can respond quickly to PWN invasion and significantly increase its expression level to generate enough defense substances to resist PWN-induced damage at 15 dpi. Although the susceptible *P. massoniana* can also respond quickly to invasion, the augmented expression of its *PmNBS-LRR97* is not enough to eliminate the damage incurred from the PWN, which can continue to impact *P. massoniana*, leaving this gene still expressed at a higher level than before the inoculation. At 30 dpi, there were almost no PWNs in the resistant *P. massoniana* due to its early resistance response to the PWN; in contrast, because the susceptible *P. massoniana* did not eliminate the PWN, resulting in wilting or even tissue death at the inoculation site, its expression level was lower than that before the inoculation. Zhang et al. [48] also found similar results in the response of grape (*Vitis quinquangularis* Rehd.) to *Plasmoparaviticola* infection. *VqCN*, a CC-NBS-LRR-type R gene, was significantly induced, and its expression level increased rapidly after the resistant variety was infected by *P. viticola*. The expression profiles of R genes in *Pennisetum glaucum* (L.) R.Br. in response to *Sclerosporagraminicola* [47], *Solanum aculeatissimum* in response to *Meloidogyne incognita* [49], and *P. monticola* in response to *Cronartiumribicola* [50] were similar to those found here for *P. massoniana*. Further, for both resistant and susceptible *P. massoniana*, *PmNBS-LRR97* was expressed more in their stems than in their needles and roots at any post-inoculation time (Figure 4B,C). This may be due to the fact that the *Monochamusalternatus* vector carrying the PWN mainly ate the stems of *P. massoniana*, enabling PWN’s entry into the host via this tissue.

Knowing the subcellular localization of genes can further help in understanding their biological functions in plants [51]. Some reported R genes locate to the nucleus [52,53,54], and others locate to the cell wall, cytoplasm [55], or cell membrane [48,56]. The PmNBS-LRR97 located exclusively to the cell membrane (Figure 4D). It could have recognized the effector proteins secreted by the PWN on the cell membrane, thus causing a defense response in the attacked *P. massoniana*.

The generation of ROS is an early event in the immune response caused by the recognition of effector proteins by the R gene, which is pivotal for inducing plant resistance responses, among which H_2_O_2_ is quite important [57,58,59,60]. NO is also an important signaling molecule that regulates plant resistance, and it is known that an increased NO concentration can enhance resistance to pathogens [61,62,63]. The contents of H_2_O_2_ and NO will both increase rapidly after a plant is attacked by pathogens. Appropriate amounts of H_2_O_2_ and NO can participate in the transmission of disease resistance signals and thereby enhance resistance. However, too much H_2_O_2_ and NO will upset the redox balance, impairing it. At this time, active oxygen-scavenging enzymes, such as SOD, POD, and CAT, are activated to remove the excess H_2_O_2_ and NO to protect plant cells from damage [64,65]. For *PmSOD*, *PmPOD*, and *PmCAT*, their respective expression patterns in the resistant and susceptible *P. massoniana* were characterized by an ‘up-down-down’ trend (Figure 6A–C). After the inoculation with the PWN, their expression levels were significantly increased at 1 dpi, but the up-regulated fold-change of resistant *P. massoniana* significantly surpassed that of susceptible *P. massoniana*. Crucially, the expression pattern of these genes was basically similar to that of *PmNBS-LRR97*, which indicates that *PmNBS-LRR97* may cause a significant increase in ROS-related genes. Next, the expression of *NbSOD*, *NbPOD*, and *NbCAT* was analyzed in the WT and transgenic tobacco, and the contents of defense signal molecules (H_2_O_2_, NO) and ROS scavenging system-related enzymes (SOD, POD, CAT) were also detected (Figure 6D–K). Evidently, the expression levels of these genes and these indicators’ contents were higher in transgenic tobacco than in WT, suggesting that the overexpression of *PmNBS-LRR97* can cause ROS production, activate enzymes that scavenge ROS, and render plants resistant to the PWN. Interestingly, its overexpression also caused tobacco’s early flowering and promoted its growth and development (Figure 5B–D). The findings can further explain the resistance conferred by *PmNBS-LRR97* to *P. massoniana* against the PWN.

## 4. Materials and Methods

### 4.1. Plant Materials and Treatment

The test materials were procured from a 5-year-old plantation of *P. massoniana* clones at the Linhai Nursery (latitude: 120°12′55.836″ E, 30°15′11.088″ N), located in Linhai City, Zhejiang Province, China. The current-year shoots of three resistant *P. massoniana* clones (all Huang 13-1) and three susceptible clones (all Guang 27-2) were selected for inoculation with the PWN in August [66]. The resistant clones were clone individuals whose growth was unaffected at 30 days post-inoculation (dpi), whereas the susceptible clones had withered and died at about 30 dpi. Each shoot was inoculated with 10,000 heads/200 μL of PWN; the counterpart control groups consisted of three selected strains of resistant and susceptible clones which received the same amount of sterile water. At 0, 1, 15, and 30 dpi with PWN, the roots, stems, and needles of the resistant and susceptible clones of *P. massoniana* in the infected and control groups were collected as three biological replicates and stored in a refrigerator at −80 °C for further study.

### 4.2. Identification and Analysis of NBS-LRR Genes in P. massoniana

These data were derived from the transcriptome data of resistant and susceptible *P. massoniana* measured by our research group in 2018 (PRJNA892753). The transcriptome materials consisted of the current-year shoots of three resistant and susceptible clones of *P. massoniana* at 0, 1, 15, and 30 dpi with PWN. At 0, 1, 15, and 30 dpi with PWN, the stems of resistant and susceptible clones of *P. massoniana* were collected as three biological replicates, and they were sent to Biomarker Technologies for transcriptome sequencing. The Hidden Markov Model of the NB-ARC conserved domain (Pfam ID: PF00931) was downloaded from the Pfam webpage (http://pfam.xfam.org/, (accessed on 5 August 2022)) [67]. To search the transcriptome protein sequence database, the hmmsearch program in HMMER v3.3 software was used (with an E-value < 1 × 10^−10^) [68], and the encountered protein sequences were screened by removing any incomplete domain sequences and redundant sequences. The retained sequences were then determined by SMART (http://smart.embl-heidelberg.de/ (accessed on 5 August 2022)) [69] and NCBI CDD (https://www.ncbi.nlm.nih.gov/Structure/cdd/wrpsb.cgi (accessed on 5 August 2022)) [70]. The LRR domain (Pfam ID: PF00560/PF07723/PF07725/PF12799/PF13306/PF13516/PF13855/PF14580) and TIR domain (Pfam ID: PF01582/PF13676.6) were identified by the same method. The CC domains were identified using the COLIS Server (https://embnet.vital-it.ch/software/COILS_form.html (accessed on 5 August 2021)).

ExPASy ProtParam (https://web.expasy.org/protparam/ (accessed on 10 August 2022)) [71] was used to predict the physicochemical properties (i.e., molecular weight, isoelectric point, instability index, aliphatic index, and grand average of hydrophilicity) of candidate proteins. Euk-mPLoc v2.0 (http://www.csbio.sjtu.edu.cn/bioinf/euk-multi-2/ (accessed on 10 August 2022)) [72] was used to predict the proteins’ subcellular localization. Signal peptides and transmembrane domains were predicted by the SignalP 5.0 Server (https://services.healthtech.dtu.dk/service.php?SignalP-5.0 (accessed on 10 August 2022)) [73] and TMHMM-2.0 (https://services.healthtech.dtu.dk/service.php?TMHMM-2.0 (accessed on 10 August 2022)) [74].

### 4.3. Phylogenetic and Conserved Motif Analysis of NBS-LRR Genes

The protein sequences containing both NBS and LRR domains were subjected to multiple sequence alignment using ClustaW [75]. Then, a phylogenetic tree was constructed using the maximum likelihood method in MEGA 7.0 [76] with these parameters: ML tree method, JTT+G+I+F model, partial deletion, and 1000 bootstrap replicates. The evolutionary tree was visualized and polished using EvolView v3 software (http://www.evolgenius.info/evolview (accessed on 13 August 2022)) [77].

Both the motif search and analysis of NBS-LRR were performed by MEME (https://meme-suite.org/meme/tools/meme (accessed on 13 August 2022)) [78] under these parameter settings: site distribution; any number of repetitions; number of motifs, 25. Finally, the protein domains and conserved motifs were visualized using TBtools v1.098765 [79].

### 4.4. Heatmap of Differentially Expressed NBS-LRR Genes

The NBS-LRR genes from resistant and susceptible P. massoniana responsive to PWN inoculation were obtained from the transcriptome data. An FDR ≤0.05 and a fold-change ≥2 were used for the identification of the differentially expressed genes (DEGs). A hierarchical Cluster heatmap based on NBS-LRR DEGs analysis was conducted with TBtools.

### 4.5. Gene Cloning and Sequence Analysis

The total RNA was extracted from the resistant and susceptible *P. massoniana* clones using an EASY38 Spin Plus plant RNA Kit (AidLab, Beijing, China), and then each RNA sample was reverse-transcribed into cDNA with the PC18-TRUEscript 1st Strand cDNA Synthesis Kit (OneStep gDNA Removal) (AidLab, Beijing, China). According to the reference sequence of *PmNBS-LRR97* in the transcriptome, Primer3 Input (https://bioinfo.ut.ee/primer3-0.4.0/ (accessed on 25 December 2022)) [80,81] was used to design the cds-specific primers (Table S1), whose PCR amplification was carried out with Phanta Max Super-Fidelity DNA Polymerase (Vazyme, Nanjing, China). The amplified sequence was ligated to the pMD20-T vector (TaKaRa, Beijing, China), transformed into DH5α *Escherichia coli*, and sequenced externally by Zhejiang Shangya Biotechnology Co., Ltd. after undergoing colony PCR.

The target sequences cloned from resistant and susceptible *P. massoniana* were aligned by DNAMAN, after which the secondary and tertiary structures of the target proteins were predicted, respectively, by SOPMA (https://npsa-prabi.ibcp.fr/cgi-bin/npsa_automat.pl?page=/NPSA/npsa_sopma.html (accessed on 20 December 2022)) [82,83] and SWISS-MODEL (https://swissmodel.expasy.org/interactive (accessed on 20 December 2022)) [84]; the corresponding phosphorylation sites were predicted using the NetPhos 3.1 Server (http://www.cbs.dtu.dk/services/NetPhos/ (accessed on 20 December 2022)) [85].

### 4.6. Real-Time Quantitative PCR (qRT-PCR) Analysis

The total RNA, which was extracted from the roots, stems, and needles of *P. massoniana* clones inoculated with the PWN and sterile water at different time points (0, 1, 15, and 30 dpi), was reverse-transcribed using PrimeScript TM RT Master Mix (TaKaRa, Beijing, China), with Primer3 Input used to design the fluorescent quantitative primers for *PmNBS-LRR97*, *PmSOD*, *PmPOD*, and *PmCAT* (Table S4) in the non-conserved domain region of the above RNA. The *EF2* of *P. massoniana* served as an internal reference, and the ABI PRISM 7300 Real-Time PCR System (Foster City, CA, USA) was operated according to the instructions of the TB Green^®^ Premix Ex Taq TM II Kit (TaKaRa, Beijing, China). Three technical replicates were performed, and the relative expression levels were calculated by applying the 2^−△△CT^ method [86] to the data. The total RNA in the leaves of wild-type (WT) and transgenic tobacco was extracted and reverse-transcribed. Using *NbACTIN* as the internal reference gene, Primer3 Input was used to design fluorescent quantitative primers for *NbSOD*, *NbPOD*, and *NbCAT* (Table S1), whose relative expression levels were calculated (as described above).

### 4.7. Subcellular Localization of PmNBS-LRR97

To clarify the subcellular localization of the PmNBS-LRR97, the full-length sequence of the gene was ligated to the pCambia1300 expression vector with GFP at its C-terminus, and this then transformed into *Agrobacterium* GV3101. Using the empty vector as a control, the *Agrobacterium* was suspended to OD_600_ = 1.2, incubated for 2 h, and then injected into the lower epidermis of tobacco leaves. Two days later, the transient expression of the GFP fusion protein was observed by the LSM900 confocal microscope imaging system (Zeiss, Oberkochen, Germany). *Nicotiana benthamiana* Domin was cultured in a light incubator at 25 °C, under a 16 h light/8 h dark cycle.

### 4.8. Generation of PmNBS-LRR97 Transgenic Lines in Tobacco

The pCambia1300-GFP expression vector containing the target gene was transformed into *Agrobacterium* GV3101, and the leaf disc method was used to transform *N. benthamiana*. In this way, the medium containing hygromycin B and PCR amplifications were performed to select the positive transgenic lines. The WT and transgenic tobacco plants’ RNA was extracted and reverse-transcribed, and the expression level of each transgenic line was analyzed by qRT-PCR, using *NbACTIN* as the internal reference gene.

### 4.9. Determination of H_2_O_2_, NO, SOD, POD, and CAT in Tobacco

The contents of H_2_O_2_, NO, superoxide dismutase (SOD), peroxidase (POD), and catalase (CAT) in 1-month-old WT and transgenic tobacco leaves were respectively determined using a hydrogen peroxide content kit (enzyme-labeled method), nitric oxide content determination kit (enzyme-labeled method), superoxide dismutase kit (WST-8 method) (enzyme-labeled method), peroxidase determination kit instruction (enzyme-labeled method), and CAT kit instruction (enzyme-labeled method), according to Suzhou Mengxi Biomedical Technology Co., Ltd.’s instructions, with three biological replicates used.

## 5. Conclusions

In this study, 507 NBS genes were identified and comprehensively analyzed in the *P. massoniana* transcriptome. The 292 NBS-LRR genes were divided into two major branches: TNL and non-TNL. *PmNBS-LRR97*, one of the important differentially expressed genes, is rapidly activated after the infection with the PWN, and its expression levels are higher in the stems than in the roots and needles of *P. massoniana*. According to its subcellular localization, PmNBS-LRR97 may recognize effector proteins on the cell membrane to elicit defense responses. *PmNBS-LRR97* may cause the significant up-regulation of ROS-related genes in *P. massoniana*. The overexpression of *PmNBS-LRR97* increases the contents of active oxygen and active oxygen scavenging enzymes in *N. benthamiana*, which may foster resistance in *P. massoniana*. Overall, these findings provide a fundamental insight into the regulation of NBS-LRR resistance genes in the defense response of *P. massoniana* to PWN.

## Figures and Tables

**Table 1:** Classification and numbers of NBS-related genes in *P. massoniana*.

Predicted Protein Domain	Letter Code	*P. massoniana*	*A. thaliana*	*P. trichocarpa*
NBS	N	90	1	53
NBS-LRR	NL	100	6	119
TIR-NBS-LRR	TNL	120	92	79
TIR-NBS	TN	68	23	13
CC-NBS-LRR	CNL	31	51	119
CC-NBS	CN	6	5	19
RPW8-NBS-LRR	RNL	41	-^a^	-
RPW8-NBS	RN	51	-	-
Other NBS		-	31	-
Total NBS genes		507	209	402

^a^—indicates that the plant species does not contain the R gene in the corresponding type.

## Data Availability

The datasets supporting the conclusions of this article are available in the NCBI Short Read Archive under accession number PRJNA892753. https://www.ncbi.nlm.nih.gov/bioproject/PRJNA892753 (accessed on 25 December 2022).

## Supplementary Materials

The following supporting information can be downloaded at: https://www.mdpi.com/article/10.3390/ijms24031812/s1.

## References

[1] Boller T., He S.Y. (2009). Innate immunity in plants: An arms race between pattern recognition receptors in plants and effectors in microbial pathogens. Science.

[2] Jones J.D.G., Dangl J.L. (2006). The plant immune system. Nature.

[3] Yuan M., Ngou B.P.M., Ding P., Xin X.F. (2021). PTI-ETI crosstalk: An integrative view of plant immunity. Curr. Opin. Plant Biol..

[4] Panstruga R., Parker J.E., Schulze-Lefert P. (2009). SnapShot: Plant immune response pathways. Cell.

[5] Ishihara T., Sato Y., Takahashi H., Uyeda I., Masuta C. (2015). Microarray analysis of R-gene-mediated resistance to viruses. Plant Virology Protocols.

[6] Kourelis J., Van D. (2018). Defended to the nines: 25 years of resistance gene cloning identifies nine mechanisms for R protein function. Plant Cell.

[7] Kourelis J., Sakai T., Adachi H., Kamoun S. (2021). RefPlantNLR: A comprehensive collection of experimentally validated plant NLRs. PLoS Biol..

[8] Bezerra-Neto J.P., Araújo F.C., Ferreira-Neto J.R.C., Silva R.L.O., Borges A.N.C., Matos M.K.S., Silva J.B., Silva M.D., Kido E.A., Benko-Iseppon A.M., Poltronieri P., Hong Y. (2020). NBS-LRR genes—Plant health sentinels: Structure, roles, evolution and biotechnological applications. Applied Plant Biotechnology for Improving Resistance to Biotic Stress.

[9] Tameling W.I.L., Vossen J.H., Albrecht M., Lengauer T., Berden J.A., Haring M.A., Cornelissen B.J.C., Takken F.L.W. (2006). Mutations in the NB-ARC domain of I-2 that impair ATP hydrolysis cause autoactivation. Plant Physiol..

[10] Ellis J.G., Dodds P.N., Lawrence G.J. (2007). Flax rust resistance gene specificity is based on direct resistance-avirulence protein interactions. Annu. Rev. Phytopathol..

[11] Meyers B.C., Kozik A., Griego A., Kuang H., Michelmore R.W. (2003). Genome-wide analysis of NBS-LRR-encoding genes in Arabidopsis. Plant Cell.

[12] Kohler A., Rinaldi C., Duplessis S., Baucher M., Geelen D., Duchaussoy F., Meyers B.C., Boerjan W., Martin F. (2008). Genome-wide identification of NBS resistance genes in *Populus trichocarpa*. Plant Mol. Biol..

[13] Christie N., Tobias P.A., Naidoo S., Külheim C. (2016). The *Eucalyptus grandis* NBS-LRR gene family: Physical clustering and expression hotspots. Front. Plant Sci..

[14] Zhou T., Wang Y., Chen J.Q., Araki H., Jing Z., Jiang K., Shen J., Tian D. (2004). Genome-wide identification of NBS genes in *japonica* rice reveals significant expansion of divergent non-TIR NBS-LRR genes. Mol. Genet. Genom..

[15] Kang Y.J., Kim K.H., Shim S., Yoon M.Y., Sun S., Kim M.Y., Van K., Lee A.H. (2012). Genome-wide mapping of NBS-LRR genes and their association with disease resistance in soybean. BMC Plant Biol..

[16] Lozano R., Ponce O., Ramirez M., Mostajo N., Orjeda G. (2012). Genome-wide identification and mapping of NBS-encoding resistance genes in *Solanum tuberosum* group phureja. PLoS ONE.

[17] Cai D., Kleine M., Kifle S., Harloff H.J., Sandal N.N., Marcker K.A., Klein-Lankhorst R.M., Salentijn E.M.J., Lange W., Stiekema W.J. (1997). Positional cloning of a gene for nematode resistance in sugar beet. Science.

[18] Song W.Y., Wang G.L., Chen L.L., Kim H.S., Pi L.Y., Holsten T., Gardner J., Wang B., Zhai W.X., Zhu L.H. (1995). A receptor kinase-like protein encoded by the rice disease resistance gene, *Xa21*. Science.

[19] Sun X., Cao Y., Yang Z., Xu C., Li X., Wang S., Zhang Q. (2004). Xa26, a gene conferring resistance to *Xanthomonas oryzae* pv. oryzae in rice, encodes an LRR receptor kinase-like protein. Plant J..

[20] Xun H., Yang X., He H., Wang M., Guo P., Wang Y., Pang J., Dong Y., Feng X., Wang S. (2019). Over-expression of *GmKR3*, a TIR-NBS-LRR type R gene, confers resistance to multiple viruses in soybean. Plant Mol. Biol..

[21] Zhao L., Sun J., Wan F., Jiang M., Zhan A. (2017). Pinewood nematode *Bursaphelenchus xylophilus* (Steiner and Buhrer) Nickel. Biological Invasions and Its Management in China.

[22] Ryss A.Y., Kulinich O.A., Sutherland J.R. (2011). Pine wilt disease: A short review of worldwide research. For. Stud. China.

[23] Zhao B.G., Futai K., Sutherland J.R., Takeuchi Y. (2008). Pine Wilt Disease.

[24] Ye J.R. (2019). Epidemic status of pine wilt disease in China and its prevention and control techniques and counter measures. Sci. Silvae Sin..

[25] Yi C.K., Byun B.H., Park J.D., Yang S.I., Chang K.H. (1989). First finding of the pine wood nematode, *Bursaphelenchus xylophilus* (Steiner et Buhrer) Nickle and its insect vector in Korea. Res. Rep. For. Res. Inst..

[26] Valadas V., Oliveira S., Espada M., Laranjo M., Barbosa P. (2012). The pine wood nematode, *Bursaphelenchus xylophilus*, in Portugal: Possible introductions and spread routes of a serious biological invasion revealed by molecular methods. Nematology.

[27] Vicente C., Espada M., Vieira P., Mota M. (2012). Pine wilt disease: A threat to European forestry. Eur. J. Plant Pathol..

[28] Abelleira A., Picoaga A., Mansilla J.P., Aguin O. (2011). Detection of *Bursaphelenchus xylophilus*, causal agent of pine wilt disease on *Pinus pinaster* in Northwestern Spain. Plant Dis..

[29] Mota M.M., Futai K., Vieira P., Ciancio A., Mukerji K. (2009). Pine wilt disease and the pinewood nematode, *Bursaphelenchus xylophilus*. Integrated Management of Fruit Crops Nematodes.

[30] Webster J., Mota M., Mota M., Vieira P. (2008). Pine wilt disease: Global issues, trade and economic impact. Pine Wilt Disease: A Worldwide Threat to Forest Ecosystems.

[31] Liu B., Liu Q., Zhou Z., Yin H., Xie Y. (2022). Overexpression of geranyl diphosphate synthase (*PmGPPS1*) boosts monoterpene and diterpene production involved in the response to pine wood nematode invasion. Tree Physiol..

[32] Liu B., Liu Q., Zhou Z., Yin H., Xie Y., Wei Y. (2021). Two terpene synthases in resistant *Pinus massoniana* contribute to defence against *Bursaphelenchus xylophilus*. Plant Cell Environ..

[33] Liu B., Xie Y., Yin H., Zhou Z., Liu Q. (2022). Identification and defensive characterization of *PmCYP720B11v2* from *Pinus massoniana*. Int. J. Mol. Sci..

[34] Custers J.H., Harrison S.J., Sela-Buurlage M.B., Van Deventer E., Lageweg W., Howe P.W., Van Der Meijs P.J., Ponstein A.S., Simons B.H., Melchers L.S. (2004). Isolation and characterisation of a class of carbohydrate oxidases from higher plants, with a role in active defence. Plant J..

[35] Fernandez-Gutierrez A., Gutierrez-Gonzalez J.J. (2021). Bioinformatic-based approaches for disease-resistance gene discovery in plants. Agronomy.

[36] Zhang Y.M., Shao Z.Q., Wang Q., Hang Y.Y., Xue J.Y., Wang B., Chen J.Q. (2016). Uncovering the dynamic evolution of nucleotide-binding site-leucine-rich repeat (NBS-LRR) genes in Brassicaceae. J. Integr. Plant Biol..

[37] Chisholm S.T., Coaker G., Day B., Staskawicz B.J. (2006). Host-microbe interactions: Shaping the evolution of the plant immune response. Cell.

[38] Arya P., Kumar G., Acharya V., Singh A.K. (2014). Genome-wide identification and expression analysis of NBS-encoding genes in *Malus x domestica* and expansion of NBS genes family in Rosaceae. PLoS ONE.

[39] Wang J., Yang C., Wu X., Wang Y., Wang B., Wu X., Lu Z., Li G. (2022). Genome-wide characterization of NBS-LRR family genes and expression analysis under powdery mildew stress in *Lagenaria siceraria*. Physiol. Mol. Plant Pathol..

[40] Alamery S., Tirnaz S., Bayer P., Tollenaere R., Chaloub B., Edwards D., Batley J. (2017). Genome-wide identification and comparative analysis of NBS-LRR resistance genes in *Brassica napus*. Crop. Pasture Sci..

[41] Mota A.P.Z., Vidigal B., Danchin E.G.J., Togawa R.C. (2018). Comparative root transcriptome of wild *Arachis* reveals NBS-LRR genes related to nematode resistance. BMC Plant Biol..

[42] Yue J.X., Meyers B.C., Chen J.Q., Tian D., Yang S. (2012). Tracing the origin and evolutionary history of plant nucleotide-binding site-leucine-rich repeat (NBSLRR) genes. New Phytol..

[43] Wu J., Zhu J., Wang L., Wang S. (2017). Genome-wide association study identifies NBS-LRR-encoding genes related with anthracnose and common bacterial blight in the common bean. Front. Plant Sci..

[44] Pendleton A.L., Smith K.E., Feau N., Martin F.M., Grigoriev I.V., Hamelin R., Nelson C.D., Burleigh J.G., Davis J.M. (2014). Duplications and losses in gene families of rust pathogens highlight putative effectors. Front. Plant Sci..

[45] Peraza-Echeverria S., Dale J.L., Harding R.M., Smith M.K., Collet C. (2008). Characterization of disease resistance gene candidates of the nucleotide binding site (NBS) type from banana and correlation of a transcriptional polymorphism with resistance to *Fusarium oxysporum* f.sp. *cubense* race 4. Mol. Breeding.

[46] Radwan O., Gandhi S., Heesacker A., Whitaker B., Taylor C., Plocik A., Kesseli R., Kozik A., Michelmore R.W., Knapp S.J. (2008). Genetic diversity and genomic distribution of homologs encoding NBS-LRR disease resistance proteins in sunflower. Mol. Genet. Genom..

[47] Veena M., Melvin P., Prabhu S.A., Shailasree S., Shetty H.S., Kini K.R. (2016). Molecular cloning of a coiled-coil-nucleotide-binding-site-leucine-rich repeat gene from pearl millet and its expression pattern in response to the downy mildew pathogen. Mol. Biol. Reps..

[48] Zhang S., Ding F., Peng H., Huang Y., Lu J. (2018). Molecular cloning of a CC-NBS-LRR gene from *Vitis quinquangularis* and its expression pattern in response to downy mildew pathogen infection. Mol. Genet. Genom..

[49] Zhou X., Liu J., Bao S., Yang Y., Zhuang Y. (2018). Molecular cloning and characterization of a wild eggplant *Solanum aculeatissimum* NBS-LRR gene, involved in plant resistance to *Meloidogyne incognita*. Int. J. Mol. Sci..

[50] Liu J.J., Xiang Y. (2019). Characterization of the western white pine TIR-NBS-LRR (*PmTNL2*) gene by transcript profiling and promoter analysis. Genome.

[51] Scott M.S., Calafell S.J., Thomas D.Y., Hallett M.T. (2005). Refining protein subcellular localization. PLoS Comput. Biol..

[52] Shen Q.H., Saijo Y., Mauch S., Biskup C., Bieri S., Keller B., Seki H., Ulker B., Somssich I.E., Schulze-Lefert P. (2007). Nuclear activity of MLA immune receptors links isolate-specific and basal disease-resistance responses. Science.

[53] Deslandes L., Rivas S. (2011). The plant cell nucleus: A true arena for the fight between plants and pathogens. Plant Signal. Behav..

[54] Bai S., Liu J., Chang C., Zhang L., Maekawa T., Wang Q., Xiao W., Liu Y., Chai J., Takken F.L.W. (2012). Structure-function analysis of barley NLR immune receptor MLA10 reveals its cell compartment specific activity in cell death and disease resistance. PLoS Pathog..

[55] He M., Xu Y., Cao J., Zhu Z., Jiao Y., Wang Y., Guan X., Yang Y., Xu W., Fu Z. (2013). Subcellular localization and functional analyses of a PR10 protein gene from *Vitis pseudoreticulata* in response to *Plasmoparaviticola* infection. Protoplasma.

[56] Qi D., DeYoung B.J., Innes R.W. (2012). Structure-function analysis of the coiled-coil and leucine-rich repeat domains of the RPS5 disease resistance protein. Plant Physiol..

[57] Lamb C., Dixon R.A. (1997). The oxidative burst in plant disease resistance. Annu. Rev. Plant Biol..

[58] Li W., Zhu Z., Chern M., Yin J., Yang C., Ran L., Cheng M., He M., Wang K., Wang J. (2017). A natural allele of a transcription factor in rice confers broad-spectrum blast resistance. Cell.

[59] Gan Y., Zhang L., Zhang Z., Dong S., Li J., Wang Y., Heng X. (2009). The LCB2 subunit of the sphingolip biosynthesis enzyme serine palmitoyltransferase can function as an attenuator of the hypersensitive response and Bax-induced cell death. New Phytol..

[60] Liu M.X., Zhang S.B., Hu J.X., Sun W.X., Padilla J., He Y.L., Li Y., Yin Z.Y., Liu X.Y., Wang W.H. (2019). Phosphorylation-guarded light-harvesting complex II contributes to broad-spectrum blast resistance in rice. Proc. Natl. Acad. Sci. USA.

[61] Lazalt A.M., Beligni M.V., Lamattina L. (1997). Nitric oxide preserves the level of chlorophyll in potato leaves infected by *Phytophthora infestans*. Eur. J. Plant Pathol..

[62] Noritake T., Kawakita K., Doke N. (1996). Nitric oxide induces phytoalexin accumulation in potato tuber tissues. Plant Cell Physiol..

[63] Durner J., Wendehenne D., Klessig D.F. (1998). Defense gene induction in tobacco by nitric oxide, cyclic GMP, and cyclic ADP-ribose. Proc. Natl. Acad. Sci. USA..

[64] Paradiso A., Caretto S., Leone A., Bove A., Nisi R., De Gara L. (2016). ROS production and scavenging under anoxia and re-oxygenation in Arabidopsis cells: A balance between redox signaling and impairment. Front. Plant Sci..

[65] Akimoto-Tomiyama C., Sakata K., Yazaki J., Nakamura K., Fujii F., Shimbo K., Yamamoto K., Sasaki T., Kishimoto N., Kikuchi S. (2003). Rice gene expression in response to N-acetylchitooligosaccharide elicitor: Comprehensive analysis by DNA microarray with randomly selected ESTs. Plant Mol. Biol..

[66] Liu Q., Wei Y., Xu L., Hao Y., Chen X., Zhou Z. (2017). Transcriptomic profiling reveals differentially expressed genes associated with pine wood nematode resistance in masson pine (*Pinus massoniana* Lamb.). Sci. Rep..

[67] Mistry J., Chuguransky S., Williams L., Qureshi M., Salazar G.A., Sonnhammer E.L.L., Tosatto S.C.E., Paladin L., Raj S., Richardson L.J. (2021). Pfam: The protein families database in 2021. Nucleic Acids Res..

[68] Eddy S.R. (2011). Accelerated profile HMM searches. PLoS Comput. Biol..

[69] Letunic I., Khedkar S., Bork P. (2021). SMART: Recent updates, new developments and status in 2020. Nucleic Acids Res..

[70] Lu S., Wang J., Chitsaz F., Derbyshire M.K., Geer R.C., Gonzales N.R., Gwadz M., Hurwitz D.I., Marchler G.H., Song J.S. (2020). CDD/SPARCLE: The conserved domain database in 2020. Nucleic Acids Res..

[71] Gasteiger E., Hoogland C., Gattiker A., Duvaud S., Wilkins M.R., Appel R.D., Bairoch A., Walker J.M. (2005). Protein identification and analysis tools on the ExPASyserver. The Proteomics Protocols Handbook.

[72] Chou K.C., Shen H.B. (2010). A new method for predicting the subcellular localization of eukaryotic proteins with both single and multiple sites: EukmPLoc 2.0. PLoS ONE.

[73] Armenteros J.J.A., Tsirigos K.D., Sønderby C.K., Petersen T.N., Winther O., Brunak S., Heijne G., Nielsen H. (2019). SignalP 5.0 improves signal peptide predictions using deep neural networks. Nat. Biotechnol..

[74] Krogh A., Larsson B., Heijne G.V., Sonnhammer E.L.L. (2001). Predicting transmembrane protein topology with a hidden Markov model: Application to complete genomes. J. Mol. Biol..

[75] Larkin M.A., Blackshields G., Brown N.P., Chenna R., McGettigan P.A., McWilliam H., Valentin F., Wallace I.M., Wilm A., Lopez R. (2007). Clustal W and Clustal X version 2.0. Bioinformatics.

[76] Kumar S., Stecher G., Tamura K. (2016). MEGA7: Molecular evolutionary genetics analysis version 7.0 for bigger datasets. Mol. Biol. Evol..

[77] Subramanian B., Gao S., Lercher M.J., Hu S., Chen W.H. (2019). Evolview v3: A webserver for visualization, annotation, and management of phylogenetic trees. Nucleic Acids Res..

[78] Bailey T.L., Johnson J., Grant C.E., Noble W.S. (2015). The MEME suite. Nucleic Acids Res..

[79] Chen C.J., Chen H., Zhang Y., Thomas H.R., Frank M.H., He Y.H., Xia R. (2020). TBtools: An integrative toolkit developed for interactive analyses of big biological data. Mol. Plant.

[80] Koressaar T., Remm M. (2007). Enhancements and modifications of primer design program Primer3. Bioinformatics.

[81] Untergasser A., Cutcutache I., Koressaar T., Ye J., Faircloth B.C., Remm M., Rozen S.G. (2012). Primer3—New capabilities and interfaces. Nucleic Acids Res..

[82] Levin J.M., Robson B., Garnier J. (1986). An algorithm for secondary structure determination in proteins based on sequence similarity. FEBS Lett..

[83] Levin J.M., Garnier J. (1988). Improvements in a secondary structure prediction method based on a search for local sequence homologies and its use as a model building tool. BBA-Protein Struct. Mol..

[84] Waterhouse A., Bertoni M., Bienert S., Studer G., Tauriello G., Gumienny R., Heer F.T., Beer T.A.P., Rempfer C., Bordoli L. (2018). SWISS-MODEL: Homology modelling of protein structures and complexes. Nucleic Acids Res..

[85] Blom N., Gammeltoft S., Brunak S. (1999). Sequence and structure-based prediction of eukaryotic protein phosphorylation sites. J. Mol. Biol..

[86] Livak K.J., Schmittgen T.D. (2001). Analysis of relative gene expression data using real-time quantitative PCR and the 2^−△△CT^ method. Methods.

