# Supplementary figures and images for: Comprehensive Analysis and Functional Verification of the Pinus massoniana NBS-LRR Gene Family Involved in the Resistance to Bursaphelenchus xylophilus
Source: Int J Mol Sci. 2023 Jan 17;24(3):1812. doi: 10.3390/ijms24031812 (PMC9915305; doi:10.3390/ijms24031812)

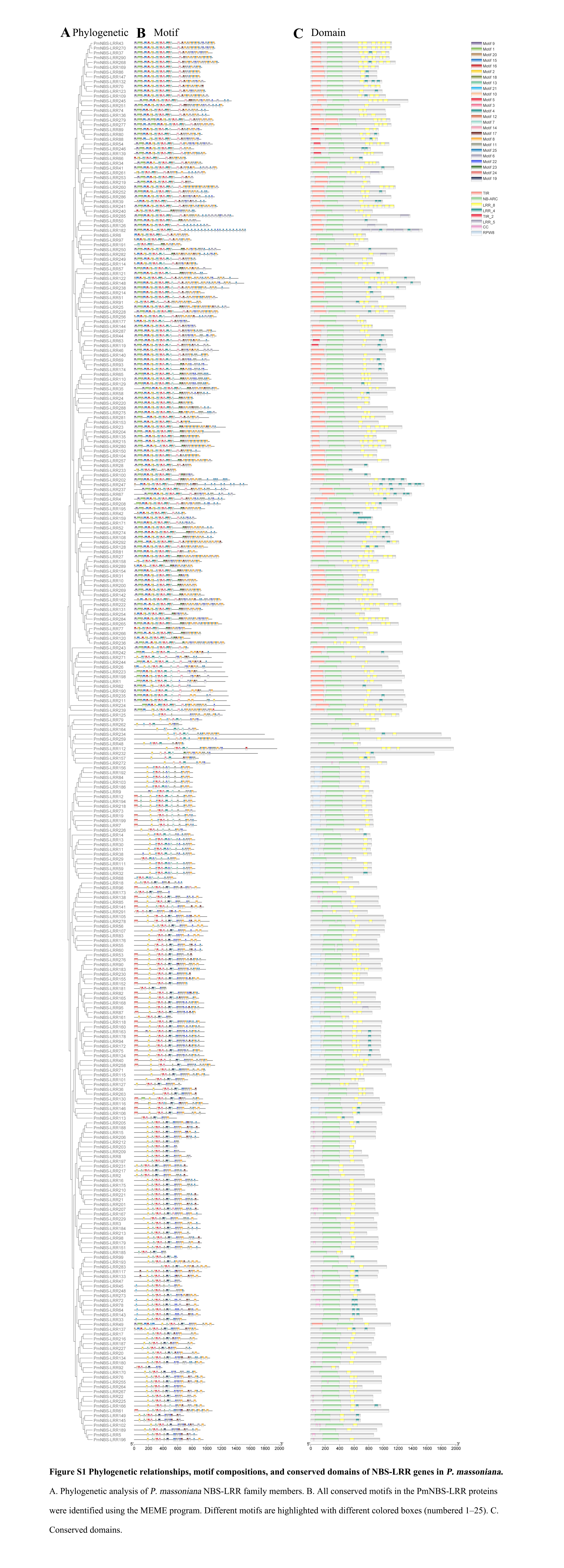

Supplement: Supplementary file 1 [file ijms-24-01812-s001.zip › Figure S1.tif]
